# Carbon nanotube dosimetry: from workplace exposure assessment to inhalation toxicology

**DOI:** 10.1186/1743-8977-10-53

**Published:** 2013-10-21

**Authors:** Aaron Erdely, Matthew Dahm, Bean T Chen, Patti C Zeidler-Erdely, Joseph E Fernback, M Eileen Birch, Douglas E Evans, Michael L Kashon, James A Deddens, Tracy Hulderman, Suzan A Bilgesu, Lori Battelli, Diane Schwegler-Berry, Howard D Leonard, Walter McKinney, David G Frazer, James M Antonini, Dale W Porter, Vincent Castranova, Mary K Schubauer-Berigan

**Affiliations:** 1 Health Effects Laboratory Division, National Institute for Occupational Safety and Health, Morgantown, WV, USA; 2 Division of Surveillance, Hazard Evaluations, and Field Studies, National Institute for Occupational Safety and Health, Cincinnati, OH, USA; 3 Division of Applied Research and Technology, National Institute for Occupational Safety and Health, Cincinnati, OH, USA; 4 NIOSH/HELD/PPRB, 1095 Willowdale Rd, MS-2015, Morgantown, WV 26505-2888, USA

**Keywords:** Workplace exposure assessment, Inhalation exposure, Mouse model, MWCNT Dose response and time dependence, Protein, Gene expression

## Abstract

**Background:**

Dosimetry for toxicology studies involving carbon nanotubes (CNT) is challenging because of a lack of detailed occupational exposure assessments. Therefore, exposure assessment findings, measuring the mass concentration of elemental carbon from personal breathing zone (PBZ) samples, from 8 U.S.-based multi-walled CNT (MWCNT) manufacturers and users were extrapolated to results of an inhalation study in mice.

**Results:**

Upon analysis, an inhalable elemental carbon mass concentration arithmetic mean of 10.6 μg/m^3^ (geometric mean 4.21 μg/m^3^) was found among workers exposed to MWCNT. The concentration equates to a deposited dose of approximately 4.07 μg/d in a human, equivalent to 2 ng/d in the mouse. For MWCNT inhalation, mice were exposed for 19 d with daily depositions of 1970 ng (equivalent to 1000 d of a human exposure; cumulative 76 yr), 197 ng (100 d; 7.6 yr), and 19.7 ng (10 d; 0.76 yr) and harvested at 0, 3, 28, and 84 d post-exposure to assess pulmonary toxicity. The high dose showed cytotoxicity and inflammation that persisted through 84 d after exposure. The middle dose had no polymorphonuclear cell influx with transient cytotoxicity. The low dose was associated with a low grade inflammatory response measured by changes in mRNA expression. Increased inflammatory proteins were present in the lavage fluid at the high and middle dose through 28 d post-exposure. Pathology, including epithelial hyperplasia and peribronchiolar inflammation, was only noted at the high dose.

**Conclusion:**

These findings showed a limited pulmonary inflammatory potential of MWCNT at levels corresponding to the average inhalable elemental carbon concentrations observed in U.S.-based CNT facilities and estimates suggest considerable years of exposure are necessary for significant pathology to occur at that level.

## Introduction

The pulmonary toxicity of carbon nanotubes (CNT) has been well described. Findings from CNT inhalation exposures included cytotoxicity, inflammatory cell influx, and interstitial fibrosis in the lung [1-6]. Some more recent studies also suggest the potential of CNT to promote lung tumorigenesis [7]. Several studies also have shown systemic effects such as immunosuppression, systemic inflammation, and changes in molecular signaling in extrapulmonary tissues [8-12]. Reduced vascular responsiveness and increased suceptibility to ischemia / reperfusion injury in cardiac tissue were also a product of CNT exposure [13,14].

In the above studies there is a lack of a correlation to occupational exposures in workers primarily due to the paucity of human exposure assessment data. This may reflect the fact that workforce sizes for CNT remain small [15]. There is little consensus on exposure assessment methods and exposure metrics (particle number, surface area, and mass) that best correlate with adverse health outcomes, although particle number is often dominated by ultrafine or non-engineered nanoparticle sources [16,17]. Traditionally, exposure assessment methods for workplace exposures to particulates have focused on the collection of gravimetric samples which has complications for particles in the ultrafine size range, though CNT occur mainly as micrometer-sized aggregates [18]. Similarly, early exposure assessment studies for CNT, which reported personal breathing zone (PBZ) samples collected within workplaces, focused on the collection of samples for the total gravimetric mass of all particles or indirectly estimated mass specific to CNT [19-21]. PBZ mass concentration ranges for these studies are summarized in Table 1 by type of exposure data collected.

**Table 1:** Studies with detectable personal breathing zone mass concentrations

**Type of samples collected**	**Personal breathing zone mass concentrations (μg/m**^**3**^**)**	**Study**
Estimated inhalable mass	0.7 - 53	Maynard et al. 2004 [21]
Total gravimetric mass	N.D. - 331.7	Han et al. 2008 [19]
Total gravimetric mass	7.8 - 320.8	Lee et al. 2010 [20]
Total carbon-inhalable size fraction	64-1094	Methner et al. 2010 [25]
Elemental carbon- inhalable size fraction	N.D. - 38	Methner et al. 2012 [24]
Elemental carbon- inhalable size fraction	N.D. - 7.86	Dahm et al. 2012 [23]
Elemental carbon- respirable size fraction	45 - 80	Birch et al. 2011 [22]

N.D. Represents non-detectable samples.

Maynard et al. collected the total mass of a metal catalyst used to produce single-walled (SW) CNT and then estimated the mass of SWCNT exposures to be between 0.7 - 53 μg/m^3^[21]. Another study conducted by Han et al. collected full-shift samples for the total mass of all particulates in a multi-walled (MW) CNT research facility and found PBZ concentrations ranging from non-detectable concentrations - 331.7 μg/m^3^[19]. The exposures at the high end were measured during blending of CNT with no control measures. Once control measures were in place, exposure was greatly diminished. Similarly, Lee et al. visited seven facilities ranging from industrial settings to lab scale settings and found full-shift total gravimetric mass concentrations from PBZ measurements to range from 7.8 - 320.8 μg/m^3^[20].

The interpretation of gravimetric sampling for high aspect ratio carbon-based nanomaterials, including CNT and carbon nanofibers (CNF), has proven difficult due to the unique characteristics of the material which includes low bulk densities and entangled / bundled structures rather than discrete fibers. Recently, several studies have utilized methodologies to measure the chemical specific mass of elemental carbon, using NMAM 5040, as a marker for CNT or CNF exposure (Table 1) [22-24]. The use of this marker has provided a more refined mass-based workplace exposure estimate, as opposed to total carbon or gravimetric dust measurements. The study by Methner et al. collected PBZ samples at a CNF end user worksite, among many other nanomaterial facilities, and measured the inhalable mass of total carbon (elemental + organic carbon), a less specific marker for CNT exposure [25]. These samples were collected during short duration exposures, aimed to identify worst-case scenarios, and found concentrations between 64 μg/m^3^ - 1094 μg/m^3^.

Subsequent studies have reported elemental carbon (EC) measurements, a more specific marker for CNT/CNF exposure. A more recent study by Methner et al. found few detectable exposures during short duration tasks at four SWCNT or CNF downstream user facilities [24]. PBZ samples were collected and analyzed for EC. One facility yielded detectable quantities of EC, which were 33 μg/m^3^ and 38 μg/m^3^. Although few of the PBZ samples had measurable EC concentrations, most of the collected samples showed evidence of SWCNT or CNF exposure by electron microscopy [24]. A study conducted by Birch et al. collected full-shift PBZ samples at a large volume CNF production facility and found concentrations at the respirable size fraction of 45 μg/m^3^ and 80 μg/m^3^[22]. Dahm et al. found patterns of exposures between primary producers and downstreams users of MWCNT, SWCNT, and CNF materials with EC exposures ranging from non-detectable concentrations to 7.86 μg/m^3^ for the inhalalable size fraction [23]. Overall, the measurement of EC is a more specific and sensitive marker of exposure which provides a more realistic workplace exposure concentration when compared to gravimetric sampling.

The occupational workforce handling CNT and CNF (beyond research and development scale) currently employs at least 500 workers at 61 companies in the U.S. with an expected growth of about 22% annually [15,26]. Currently there are no known end-point effects in humans following CNT exposure. This leads to extrapolation from rodent studies. Given the toxicity observed in rodents, epidemiologic studies have been initiated world-wide [26]. One key question becomes obvious: how do realistic U.S. workplace exposures relate to the toxicity found in animal studies? The present study was designed to address this question. The goal was to expose animals to a high dose that would cause significant inflammation with histological findings and then a low dose to serve as a no observable effect level. This design will serve as a reference for detailed molecular analysis, pulmonary pathology, systemic inflammation, and evaluation of cardiovascular dysfunction at human relevant exposures.

The inhalation study utilized the MWCNT produced by Hodogaya, commonly referred to as the Mitsui MWCNT or MWNT-7. This particular product was utilized for several reasons: 1.) A majority of the U.S. workforce handling carbonaceous nanomaterials primarily produces or utilizes MWCNT [15]. 2.) Economically, the global market showed that CNT represents 28% of the total engineered nanomaterial market share with MWCNT being 94% of the total CNT production value (http://www.nanowerk.com/spotlight/spotid=23118.php). 3.) The Hodogaya MWCNT has been extensively characterized and pulmonary effects are known for certain lung burdens [5,27]. 4.) Ongoing studies are obtaining and evaluating specific CNT materials that are being used primarily in U.S. facilities. Results for the Hodogaya-produced MWCNT will permit comparison with products utilized by U.S. facilities.

## Results and discussion

### Exposure assessment

#### PBZ elemental carbon measurements in MWCNT facilities

**Figure 1:**
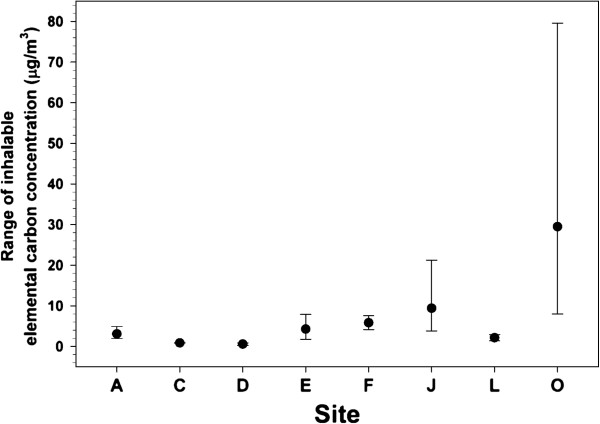
**Inhalable elemental carbon (EC) concentrations at eight MWCNT facilities.** The mean, with error bars representing the upper and lower range, of measured EC concentration (μg/m^3^) with background correction. The figure was adapted from data previously published, sites A, C-F [23], while including 3 additional sites, L, J, and O. The studied sites were identified in a manner to be consistent with nomenclature of specific sites. For example, site B was a single-walled carbon nanotube facility and therefore excluded.

The range of inhalable EC concentrations from PBZ samples at 8 facilites handling MWCNT are shown in Figure 1. The figure shows the arithmetic mean exposure concentrations with background EC correction for each site with the error bars indicating the upper and lower range of measured exposures. The first 5 sites (A,C,D,E, and F; Site B was excluded due to the production of SWCNT), were adapted from Dahm et al. [23]. As part of an ongoing study, an additional 3 MWCNT facilities have been assessed. These companies produce, utilize, and handle the material throughout the workday as well and have been added to the existing data.

The average EC concentrations at the inhalable size fraction from the eight total MWCNT sites was found to have an arithmetic mean of 10.6 μg/m^3^ with a standard deviation of 17.2 (geometric mean- 4.21 μg/m^3^ and geometric standard deviation of 4.15). In these 8 MWCNT facilities, exposures ranged from non-detectable samples to 79.6 μg/m^3^ and the exposure levels were log normally distributed (Shapiro-Wilk p=0.97). Nearly all of the samples that were found to be > 10 μg/m^3^ came from a single facility; however, the number of PBZ samples collected at this site was representative of the facility’s percentage of the total MWCNT-exposed workforce. Thus, the reported distribution of exposure levels should reflect the target population of workers.

The NIOSH Current Intelligence Bulletin on CNT and CNF has recently published a recommended exposure limit (REL) of 1 μg/m^3^ for an 8 h time-weighted average of EC [28]. The REL is for the respirable fraction of CNT. The data discussed in the above results is for the inhalable fraction. A recently study on the dustiness of various types of nanomaterials provided the respirable to total or inhalable size fraction ratio [29]. Only one MWCNT and one CNF were reported. Ratios of 0.17 and 0.28 respirable to total were found, respectively. It is anticipated that different types of CNT powders may behave differently and the ratio of respirable to inhalable or total may vary depending on the material handled, the dust generation process, the aerosol concentration, and the sampling location. Continuing studies within nanomaterial facilities are routinely collecting PBZ respirable fractions for MWCNTs. Preliminary results indicate similar findings to those predicted by dustiness studies. Therefore, assuming a 25% respirable fraction, the respirable fraction as an average of all 8 facilites would be approximately 2.65 μg/m^3^. This is in sharp contrast to 45 and 80 μg/m^3^ of respirable EC found at a CNF facility [22]. However, this facility produced large quantities of material and was using inadequate exposure control strategies. Given that 75% of MWCNT exposure measurements for inhalable EC were < 10 μg/m^3^, it would suggest that exposures to CNT can be contained with proper engineering controls [19,23]. However, it should be noted that the possible health effects of exposure to inhalable (but non-respirable) MWCNT are unknown.

### Images of workplace exposures

**Figure 2:**
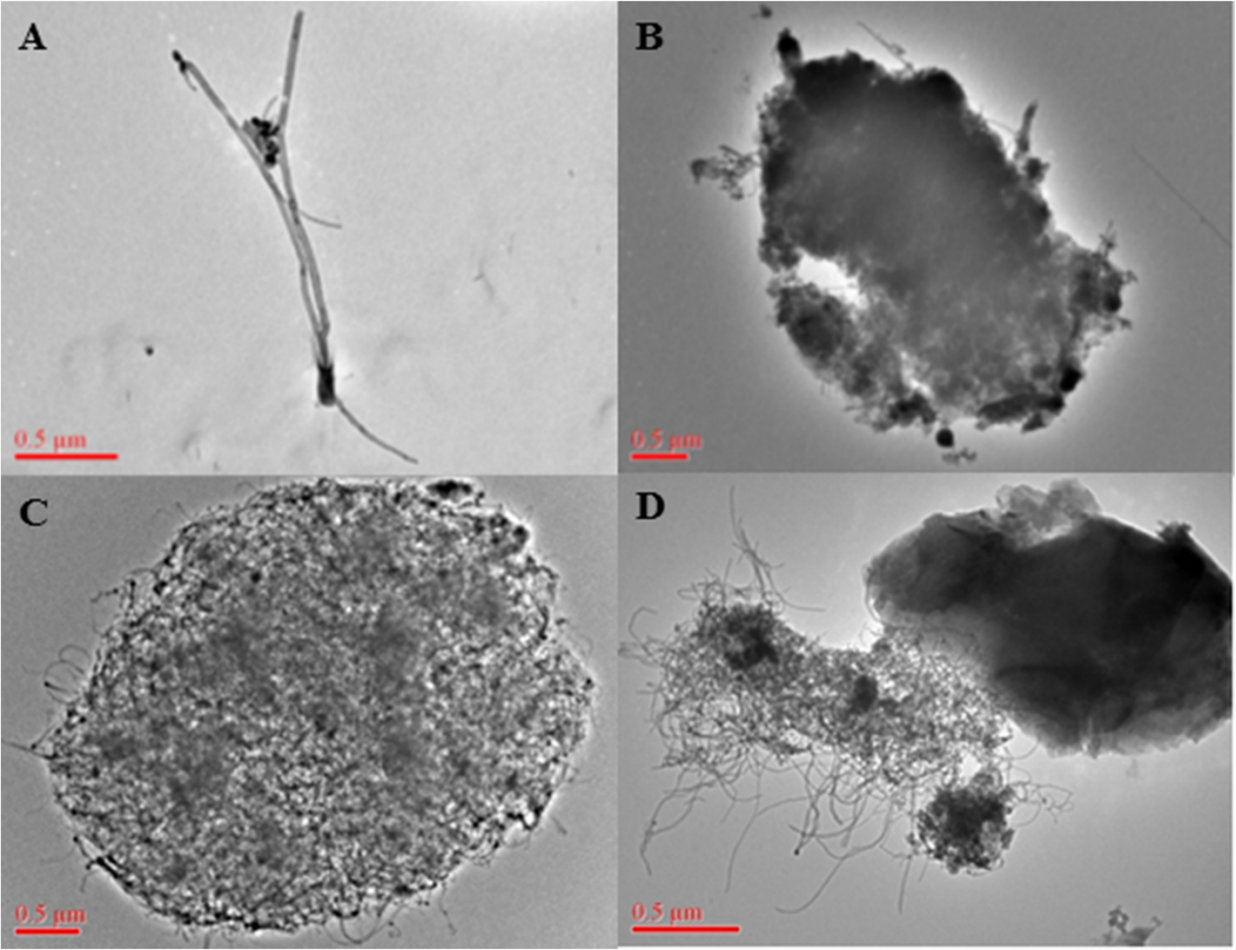
**Images of multi-walled carbon nanotubes collected from facilities.** Electron microscopy of filters collected from personal breathing zone sampling showed exposures can range from dispersed panel **(A)** to more agglomerated panel **(B-D)**.

In addition to the measure of EC concentrations, representative electron microscopy images of samples collected from MWCNT workplaces were evaluated. Figure 2 shows the variety of particle types that can be found in facilities handling MWCNT. In rare instances, the MWCNT aerosol can be partially comprised of particles resembling single fibers or bundles of only a few fibers, Figure 2A. However, it is much more common to find an aerosol comprised of tangled, agglomerated material several μm in diameter, Figure 2B-D. Similar findings of both dispersed and tangled CNT and CNF materials can be found from electron microscopy results from other studies [19,20,23-25]. These differences can be related to the physical and chemical characteristics of the type of high aspect ratio carbon-based nanomaterials being produced as well as the particular handling process in the facility. These images illustrate the complexity when designing an *in vivo* study to mimic the workplace. Data suggest the degree of dispersion is related to the fibrotic potential of CNT [30]. In addition, a more aggregated SWCNT produced less pulmonary and systemic inflammation compared to a more dispersed MWCNT at an equal mass dose [8]. Therefore, given the vast diversity of characteristics of CNT products, coupled with varying handling processes, animal exposures to agglomerated and dispersed CNT have relevancy to the occupational setting.

### Extrapolation of lifetime cumulative worker exposures to rodent dosimetry

Although the worker exposure data were well described by a lognormal distribution, the arithmetic mean (rather than the geometric mean) was used to estimate the average cumulative working lifetime exposure in humans, because the arithmetic mean (unlike the geometric mean) sums to the appropriate cumulative total over a time period and/or population [31]. For this reason, the arithmetic mean is often preferred over the geometric mean in epidemiologic studies (e.g. [31,32]). The extrapolation to murine equivalence was based on standard worker ventilatory parameters (31% sitting, 69% light exercise with a minute ventilation of 20 L/min [33]) of a worker being exposed to 10.6 μg/m^3^, the average inhalable EC concentration of MWCNT measured at 8 U. S. facilities. The alveolar deposition expected in a worker exposure was estimated. The alveolar deposition will be dependent on the mass median aerodynamic diameter (MMAD). Dustiness studies showed 0.17 and 0.28 of the inhalable fraction of MWCNT and CNF to be respirable [29]. Using the ACGIH sampling criteria for inhalable and respirable fractions, a 25% respirable fraction would approximate to a MMAD of roughly 5.5 μm. Human alveolar deposition fraction, assuming a monodispersed aerosol and a single mode distribution, for a MMAD of 5.5 μm was estimated to be 4% [33,34]. Therefore, alveolar deposition in the worker was calculated as follows:

$$\begin{array}{ll} \text{Human} & :\;\text{Airborne Conc}\\ & \;\times V_{E}\;\text{x exposure duration}\\ & \;\times \text{alveolar deposition efficiency}\\ & =\text{human alveolar deposition} \end{array}$$

$$\begin{array}{ll} \text{For}\;10.6\;\mathrm{\mu g}/m^{3} & :\;\left(10.6\;\mathrm{\mu g}/m^{3}\right)\times \left(20L/\text{min}\right)\\ & \;\times \left(10^{-3}m^{3}/L\right)\times \left(8\text{hr}/d\right)\\ & \;\times \left(60\text{min}/h\right)\times 4\%\\ & =4.07\;\mathrm{\mu g}/d\;\text{alveolar deposition} \end{array}$$

The extrapolation showed that a worker exposed to an inhalable EC concentration of 10.6 μg/m^3^ for an 8 hour day can roughly expect an approximate alveolar depisition of 4.07 μg/d assuming a MMAD of 5.5 μm. This deposition was then converted to mouse equivalence by alveolar surface area [35]:

$$\begin{array}{l} \text{Human Deposition}/\text{Human Alveolar Surface Area}\\ \;=\mathrm{Mouse Deposition}/\text{Mouse Alveolar Surface Area}\frac{4.07\mathrm{\mu g}/d}{102\;m^{2}}\\ \;=\frac{\left(x\right)}{0.05\;m^{2}}\mathrm{Mouse equivalent alveolar deposition}=x=2\text{ng}/d \end{array}$$

Therefore, in a mouse this worker exposure is equivalent to 2 ng/d.

Using previously determined alveolar depositions (detailed in the Methods), mice were exposed to MWCNT by inhalation for 4 weeks (19 d of inhalation over 26 d) to deliver an alveolar lung burden of 1970 ng/d, 197 ng/d, or 19.7 ng/d (Table 2). The daily deposition in the low dose group represents approximately 10 d of a human exposed to an inhalable concentration of 10.6 μg/m^3^, the middle dose 100 d, and the highest dose 1000 d. Therefore, our cumulative 19 d deposition using a standard 5 day workweek for 250 d/yr for an individual exposed to an inhalable EC concentration of 10.6 μg/m^3^ was 190 d (0.76 yr), 1900 d (7.6 yr), or 19,000 d (76 yr) for the low, middle, and high dose respectively (Table 2).

**Table 2:** Predicted lung burden and relationship to human equivalence

**Exposure**	**Total lung burden per day (ng)**	**Alveolar lung burden per day (ng)**	**Total alveolar burden (μg)**	**Alveolar lung burden compared to human exposed at 10 μg/m**^**3 **^**for 8 h**
				**Daily (cumulative)**
5 mg/m^3^ for 5 h/d for 19 d	2340	1970	37.43	1000 days (76 years)
0.5 mg/m^3^ for 5 h/d for 19 d	234	197	3.74	100 days (7.6 years)
0.5 mg/m^3^ for 0.5 h/d for 19 d*	23.4	19.7	0.37	10 days (0.76 years)

*Mice were exposed to air for 4.5 h prior to 0.5 h of MWCNT to maintain consistency of total exposure time.

Caveats of the above extrapolation were the MMAD of 5.5 μm and utilization of mass as the dose metric. As shown in Figure 2, the potential exists for a range of dispersions in the facility which would affect the MMAD and, therefore, the alveolar deposition. The inhalation system developed at NIOSH for *in vivo* testing dispersed the MWCNT used in this study to a MMAD of 1.5 μm [27]. If a MMAD of 1.5 μm was observed in a MWCNT facility, then the estimated alveolar deposition would increase from 4% to 11% [33,34]. Extrapolating 11% alveolar deposition, the inhalation design of this study would represent 0.27, 2.7, and 27 years of a human exposed to an inhalable EC concentration of 10.6 μg/m^3^. Lastly, extrapolation of effects in this study assumes complete exposure and does not take into account respirator protection. The correct and properly fitted respirator would protect according to the assigned protection factor.

The exposure assessment data and extrapolations from this study were done using mass-based analysis as opposed to fiber number (e.g. nanotubes/cm^3^). Currently, NIOSH is attempting to develop methodologies utilizing controlled inhalation exposures to MWCNT to accurately assess the fiber number in the aerosol [27] and such fiber count methodologies are being explored from the collected human PBZ from this study. Even when counting methodologies are utilized, it will be difficult to link directly to end-point pathology as degree of dispersion can affect pulmonary fibrosis and recent evidence suggests the potential for larger agglomerates to dissociate into smaller structures or singlets over time [30]. Difficulties will also arise based on the type of CNT material being produced and handling procedures which will alter the agglomerate characteristics and potentially the pathology. Given the current REL for CNT of 1 μg/m^3^ is a mass-based measurement [28], extrapolations from this study utilized mass.

### Inhalation toxicology

#### In vivo inhalation parameters

**Figure 3:**
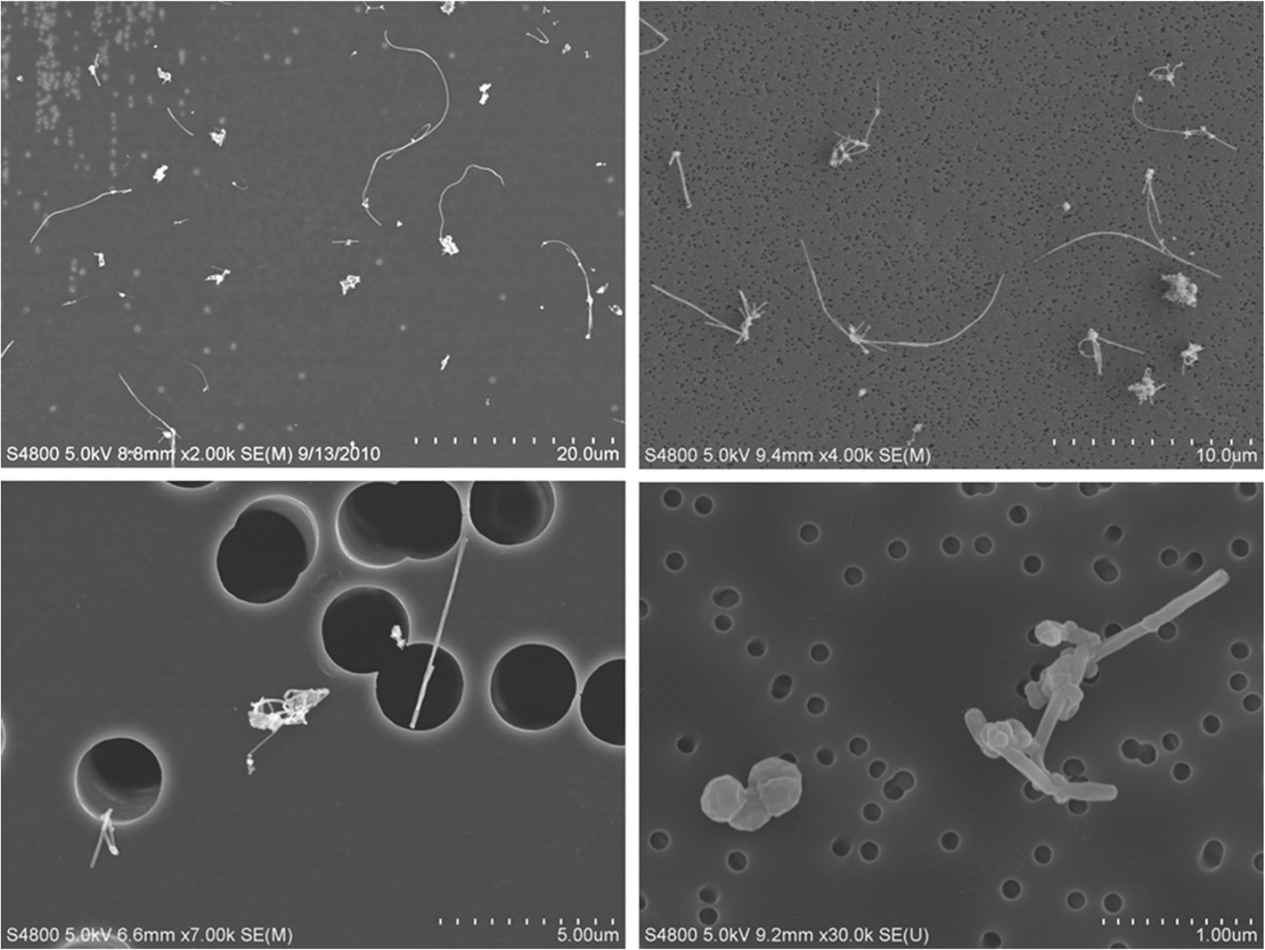
**Images of multi-walled carbon nanotubes (MWCNT) from the rodent inhalation study.** Scanning electron microscopy images of MWCNT collected on the most populated stage of a Nano MOUDI, showing a well-dispersed material. The images represent four different magnifications indicated by the scale bars (20, 10, 5, and 1 um). The 5 μm scale bar had a pore size (dark circles) of 2.5 μm and the other three figures had a pore size of 0.1 μm. The mass median aerodynamic diameter of the material was 1.5 μm. The count mode aerodynamic diameter was ~400 nm.

**Figure 4:**
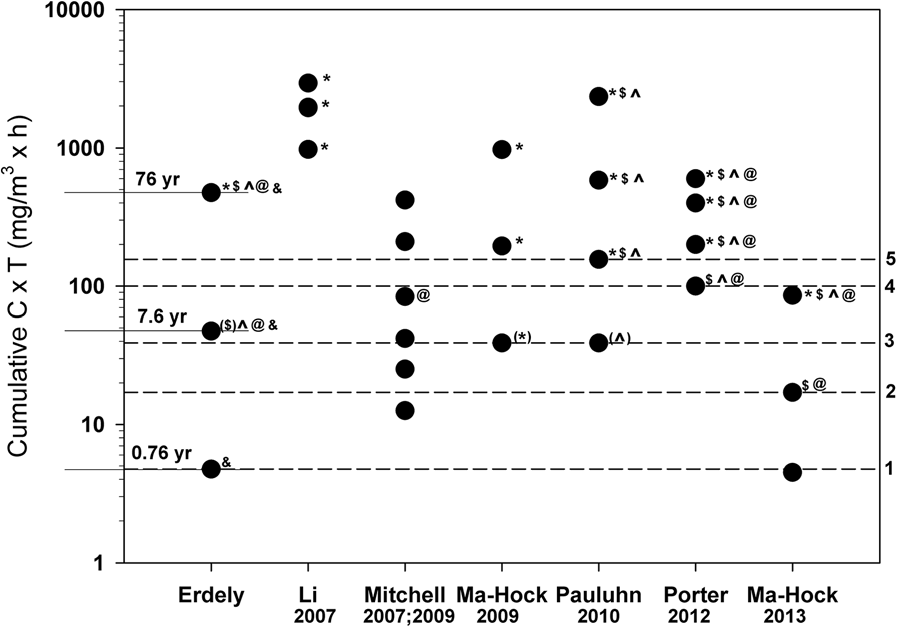
**Comparison of effects among studies of inhalation exposure to multi-walled carbon nanotubes (MWCNT).** Cumulative MWCNT exposures (concentration (C) x time (T) – mg/m^3^ multiplied by total hours of exposure) were plotted on a logarithmic scale from studies that exposed rodents by inhalation to various doses of MWCNT (black circles). Measured toxicologic endpoints were identified for each cumulative exposure. Symbols represent histopathologic alterations (*), increased bronchoalveolar lavage (BAL) polymorphonuclear cell (PMN) influx or enzyme activity ($), increased BAL total protein or albumin (^), increased tissue or BAL cytokines (@), and alterations in mRNA expression (&). The lack of a symbol means either the endpoint was not measured or there was no effect due to treatment. Symbols in parantheses indicate either a transient effect, the effect was scored as minimal, or the response was not consistently measured at all post-exposure time points for that given cumulative C x T. Each of the 5 toxicologic endpoints were highlighted by an arbitrary cutoff (dashed lines) to illustrate the threshold cumulative C x T to result in significant pulmonary responses in general agreement across various study designs. The numbers representing the dashed lines are as follows: 5 – histological alterations, 4 – increased BAL PMN influx or enzyme activity, 3 – increased BAL total protein or albumin, 2 – increased tissue or BAL cytokines, and 1 – alterations in mRNA expression. The years indicated on the left side of the graph are the extrapolations of the mouse lung burden from this study to represent years of worker exposure to an inhalable elemental carbon concentration of 10.6 μg/m^3^ assuming 25% respirability which predicts a MMAD of 5.5 μm.

Representative SEM images of aerosolized MWCNT collected from the most populated stage of a Nano MOUDI from the rodent inhalation study indicated a well dispersed material (Figure 3). The mass median aerodynamic diameter was 1.50 μm with a geometric standard deviation of 1.67. The count mode aerodynamic diameter was ~400 nm. In addition, there were no differences in inhalation parameters when comparing 5 mg/m^3^ to 0.5 mg/m^3^. The summary of inhalation parameters showed that the cumulative target doses of 475, 47.5, and 4.75 mg/m^3^ x h were obtained during the 19 d inhalation design (Table 3). Figure 4 shows a comparison of this design to 7 other inhalation dose–response studies utilizing MWCNT by cumulative concentration multiplied by time (C x T) [1-5,10,11]. The studies by Li et al. and Porter et al. varied the days of exposure while keeping the concentration constant [1,5]. The high dose group was exposed at a daily rate consistent with high dose exposures of previous studies with a cumulative C x T expected to cause inflammation and histological changes (Figure 4). The middle dose provided a cumulative deposition comparative to the lowest doses used in the 13 week inhalation studies by Pauluhn and Ma-Hock et al. [3,4]. The low dose was the same as a recently published study with expectations of a no effect level [2].

**Table 3:** Summary of inhalation exposure parameters

	**Daily exposure concentration and duration**				Exposure dose*
**Experiment**	**Mean concentration (mg/m**^**3**^**)**	**RSD (%)**	**Daily mean exposure (min)**	**RSD (%)**	**C x T (mg/m**^**3 **^**x h)**
**1 – High dose**	4.67	7.8	321	1.3	475.4
**2 – Middle dose**	0.49	6.3	309	2.1	47.6
**3 – Low dose**	0.32	6.8	48	10.6	4.8
**4 – High dose**	4.68	9.4	320	2.2	475.4
**5 – Middle dose**	0.48	5.7	309	2.4	47.5
**6 – Low dose**	0.42	6.5	36	10.5	4.7

*RSD* Relative standard deviation.

*Exposure for 19 day.

#### Pulmonary response to MWCNT inhalation exposure

**Figure 5:**
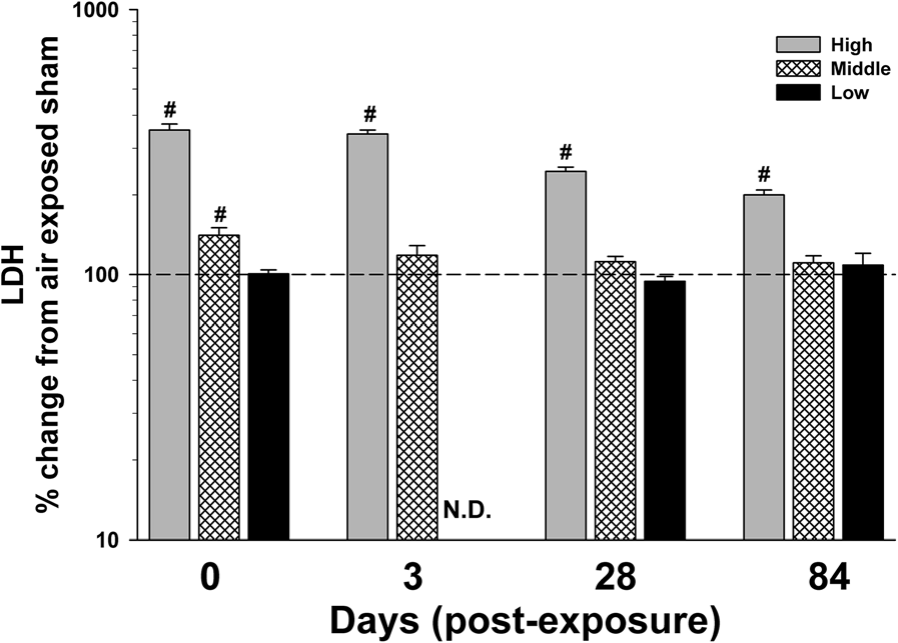
**Effect of multi-walled carbon nanotube (MWCNT) inhalation on pulmonary cytotoxicity at different time points after exposure.** Lactate dehydrogenase (LDH) activity (U/L), an indication of cytotoxicity, was measured from the first fraction of collected bronchoalveolar lavage fluid. Data are expressed as percent (%) change from respective sham (dashed line – 100%). N.D. – not determined. #p<0.05 vs. other MWCNT depositions for a given time point only.

**Figure 6:**
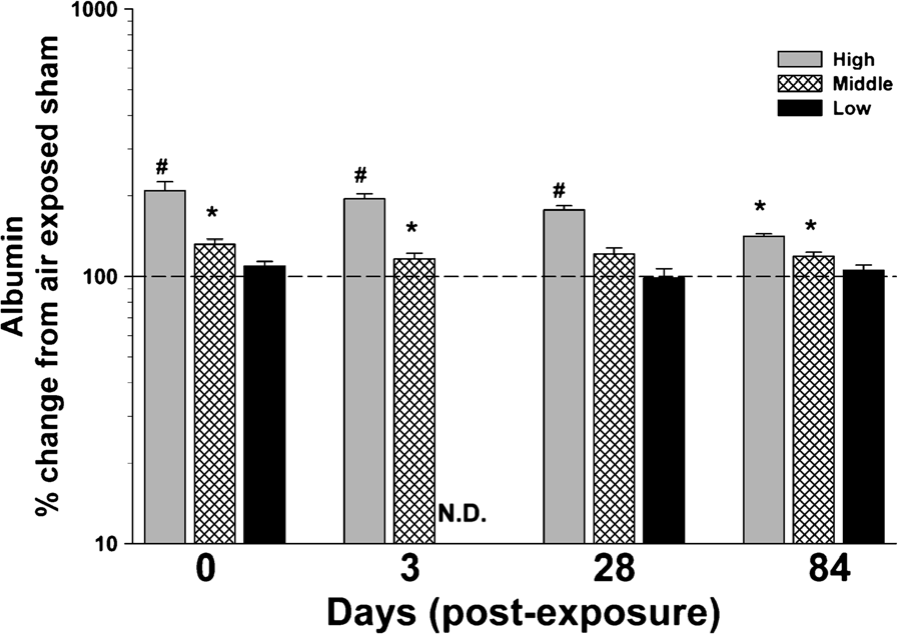
**Effect of multi-walled carbon nanotube (MWCNT) inhalation on pulmonary permeability at different time points after exposure.** Albumin (mg/dL), an indication of a loss of epithelial barrier integrity, was measured from the first fraction of collected bronchoalveolar lavage fluid. Data are expressed as percent (%) change from respective sham (dashed line – 100%). N.D. – not determined. *p<0.05 vs. respective sham; #p<0.05 vs. other MWCNT depositions for a given time point only.

The high dose of MWCNT inhalation exposure, as expected, resulted in marked cytoxicity, as measured by bronchoalveolar lavage (BAL) lactate dehydrogenase (LDH) activity, that was maintained 84 d post-exposure (Figure 5). The middle dose resulted in transient cytotoxicity that returned to baseline by 3 d. There was no cytotoxicity measured at the lowest lung burden. Loss of epithelial barrier integrity, measured as BAL albumin levels, was significant through 84 d post exposure to the high dose of MWCNT (Figure 6). The middle dose also showed increased levels of albumin that persisted through 84 d post-exposure. There was no effect at the low dose.

**Figure 7:**
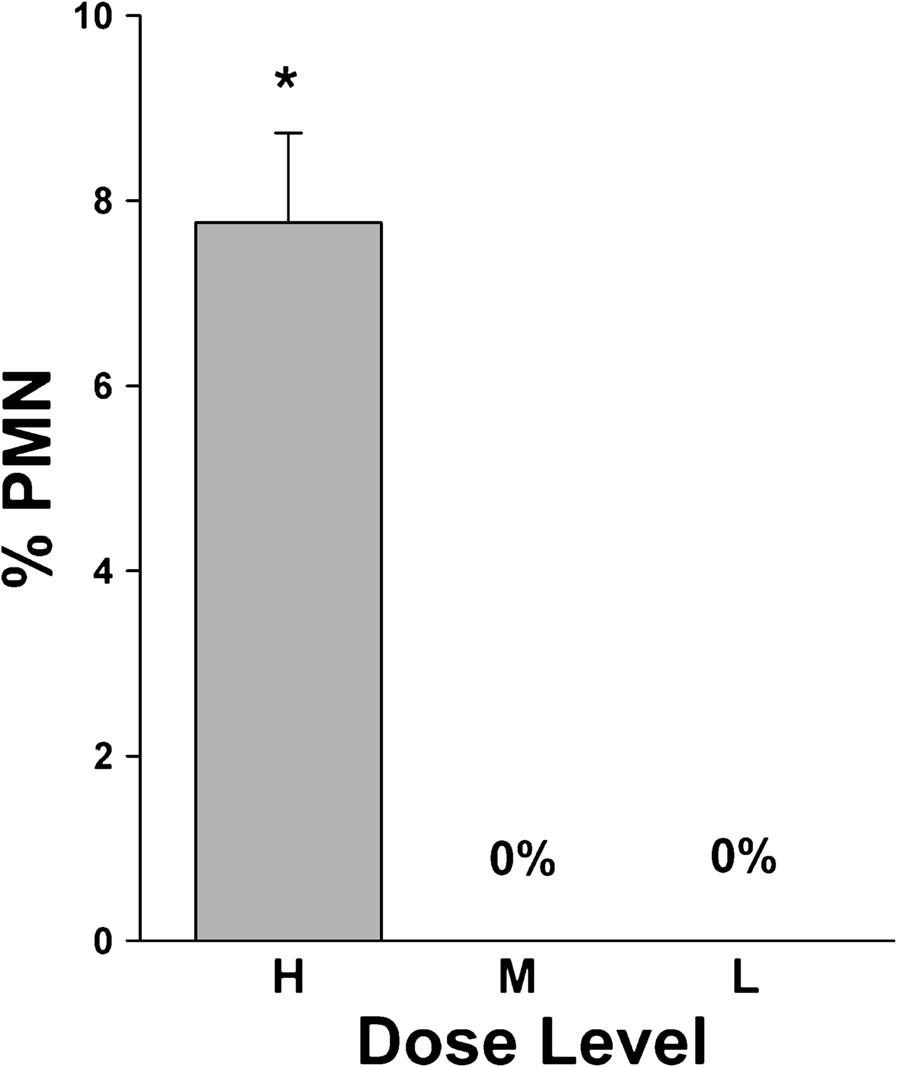
**Effect of multi-walled carbon nanotube (MWCNT) inhalation on pulmonary inflammatory cell influx at 0 d post-exposure.** Polymorphonuclear cells, an indication of pulmonary inflammation, were measured from reconstituted pellets of the bronchoalveolar cellular fraction. H = high dose; M = middle dose; L = low dose. Data are expressed as percent (%) PMN from a total of 300 cells counted slides from cytospins. *p<0.05 vs. respective sham.

The middle inhalation dose did not result in any significant polymorphonuclear (PMN) cell influx (Figure 7). These data suggest that exposures to purified MWCNT at dose levels relevant to occupational exposures do not elicit a PMN influx. From an extrapolated dosimetry argument, PMN accumulation is not likely a major factor in the occupational setting without considerable accumulation or inflammatory contamination (e.g. catalytic metals, endotoxin). The high dose showed a significant PMN influx of 7.7% at 0 d (Figure 7) with significant levels maintained through 84 d (data not shown). The % of PMN is in sharp contrast to our previous work showing that 4 h after a 40 μg bolus dose of MWCNT the differential % of PMN was 55% [8]. It is unclear the impact of the lack of a PMN response at human relevant exposures. Previous studies have shown that peroxidases produced by PMN could facilitate the degradation of CNT [36,37]. The loss of that potential degradation mechanism and reduced overall inflammation may affect the life cycle of MWCNT in the lung. Ongoing quantitative studies are assessing the linearity of pulmonary distribution and fate of MWCNT from the current time course and dose–response study.

**Figure 8:**
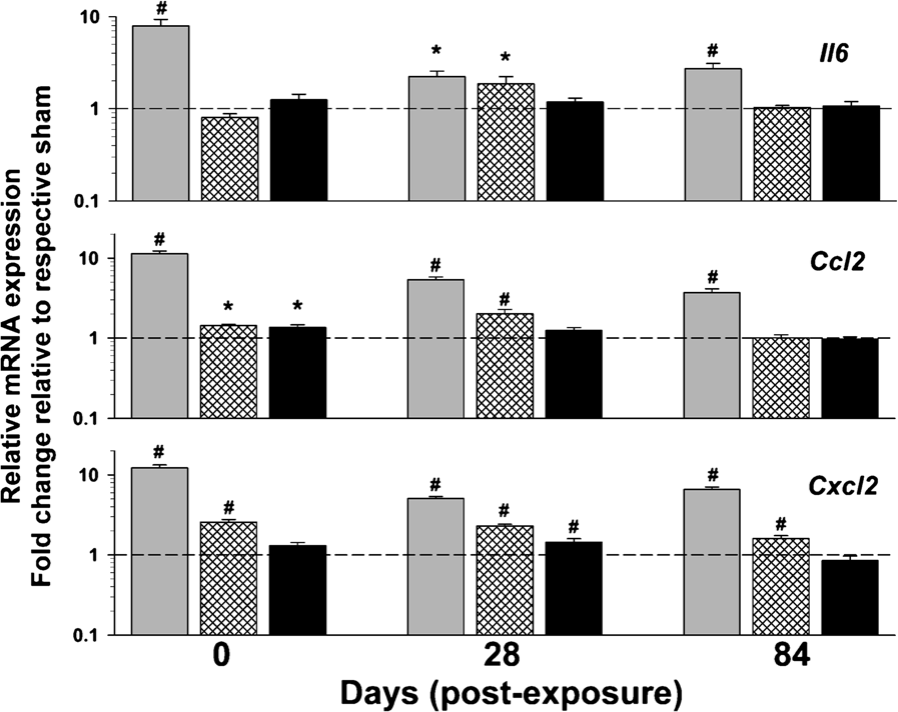
**Effect of multi-walled carbon nanotube (MWCNT) inhalation on pulmonary inflammatory gene expression at different time points after exposure.** Interleukin 6 (*Il6*), chemokine (C-C motif) ligand 2 (*Ccl2*; also referred to as monocyte chemotactic protein-1), and chemokine (C-X-C motif) ligand 2 (*Cxcl2*; also referred to as macrophage inflammatory protein 2-alpha) were measured. Data are expressed as percent (%) change from respective sham (dashed line – 100%). Data presented as high dose (gray bars), medium dose (pattern), and low dose (black bars). *p<0.05 vs. respective sham; #p<0.05 vs. other MWCNT depositions for a given time point only.

**Figure 9:**
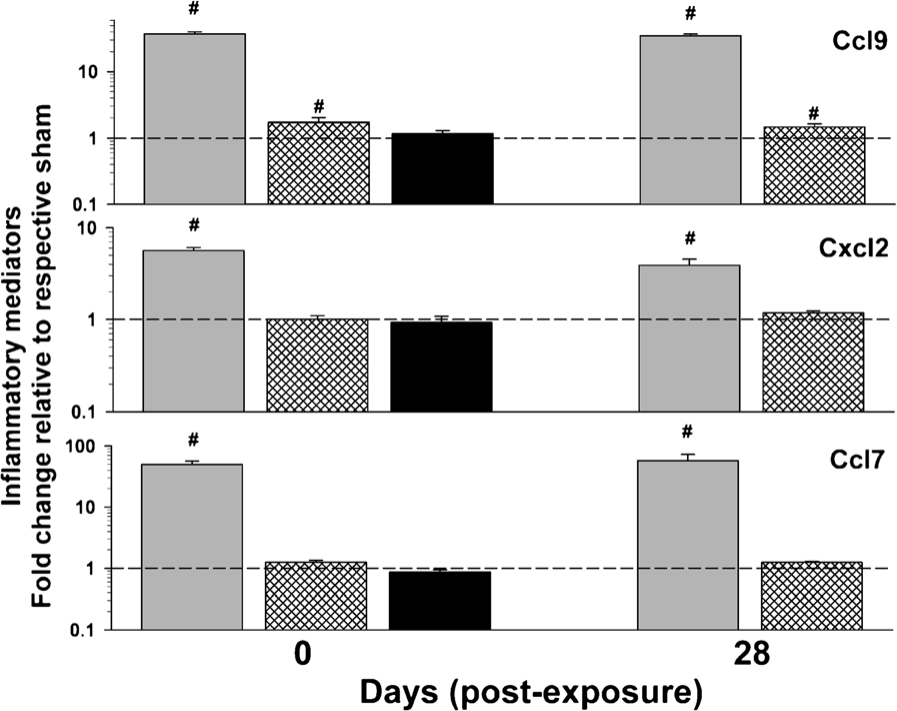
**Effect of multi-walled carbon nanotube (MWCNT) inhalation on pulmonary inflammatory protein levels at different time points after exposure.** Chemokine (C-X-C motif) ligand 2 (Cxcl2), chemokine (C-C motif) ligand 7 (Ccl7), and chemokine (C-C motif) ligand 9 (Ccl9) were measured from the first fraction of collected bronchoalveolar lavage fluid. Data are expressed as percent (%) change from respective sham (dashed line – 100%). Measurements were made from all doses at 0 d and the high and middle dose at 28 d since no effect was observed in the low dose at 0 d. Data presented as high dose (gray bars), medium dose (pattern), and low dose (black bars). *p<0.05 vs. respective sham; #p<0.05 vs. other MWCNT depositions for a given time point only.

Additional measures of inflammation, including tissue mRNA expression changes and BAL mediator levels, were measured. As expected, levels of mRNA expression in the lung of several classic inflammatory mediators, including interleukin 6 (*Il6*), chemokine (C-C motif) ligand 2 **(***Ccl2*; also referred to as monocyte chemotactic protein-1), and chemokine (C-X-C motif) ligand 2 (*Cxcl2*; also referred to as macrophage inflammatory protein 2-alpha), were increased through 84 d following the high dose exposure (Figure 8). There were also effects at the middle dose for all 3 genes and at the low dose for *Ccl2* and *Cxcl2* (Figure 8). BAL fluid protein levels of Il-6 and Ccl2 were increased at the high dose but did not reach the level of detection using multiplex technology for the middle and low doses, so it is unclear beyond the high dose whether increased transcription contributes to a corresponding increase in protein levels (data not shown). Conversely, BAL protein levels for Cxcl2, chemokine (C-C motif) ligand 7 (Ccl7; also known as monocyte-specific chemokine 3), and chemokine (C-C motif) ligand 9 (Ccl9; also known as macrophage inflammatory protein-1 gamma) were increased (Figure 9), indicating the presence of inflammatory mediators at least 28 d post-exposure to the middle dose.

The BAL inflammatory findings detailed in this study qualitatively agree with those of other MWCNT inhalation studies (Figure 4). Porter et al. reported cytotoxicity and increased PMN at the lowest cumulative C x T exposure dose of 100 mg/m^3^ x h. [5]. Studies using lower cumulative exposures showed no cytotoxicity or PMN influx [4,10]. In this study, there was a transient increase in cytotoxicity at 0 d in the middle dose. This indicates that 19 d inhalation of 0.5 mg/m^3^ for 5 h/d is cytotoxic but the deposition level of 197 ng/d did not sustain the response for the particular MWCNT studied. Changes in BAL total protein or albumin appears to have a lower threshold. The middle dose in this study as well as the low dose in the Pauluhn study indicated increased BAL albumin and total protein, respectively [4]. Increased BAL albumin levels without cytotoxcity have been observed following other particulate exposures [38]. As should be expected, not all studies are in perfect agreement with the arbitrary cutoffs in Figure 4. In particular, a recent study by Ma-Hock et al. showed significant cytotoxicity at lower C x T than the transient response of the middle dose from this study without increased total protein in the BAL [2]. Increased inflammatory proteins were measured in the BAL at the middle dose in the panel analyzed; this is in agreement with previous studies [5,11] as well as the middle dose by Ma-Hock et al. [2]. These effects also tended to be below the threshold for marked cytotoxicity or PMN influx. Lastly, increased transcription of inflammatory markers was evident at the lowest cumulative C x T (Figure 4). Although increased protein levels were not evident, these data show a low level of underlying inflammatory signaling at lung burdens which do not cause any measurable toxicity. The increased mRNA expression may be in response to the process of pulmonary handling of the daily nanogram levels of deposited MWCNT. The genes measured in Figure 8 are either produced by or enhance the accumulation of macrophages. The MWCNT used in this study were primarily found in alveolar macrophages following exposure supporting macrophage-related signaling [39]. Ongoing studies are using global mRNA expression profiling by microarray paired with subsequent pathway analysis to compare and contrast response mechanisms associated with a high dose exposure and a lung burden more relevant to the workplace. Given that transcriptional changes were evident at a 10 times lower deposition than necessary to induce more traditional markers of pulmonary toxicity, the approach seems logical. Many of the human analogs for these gene expression products are being analyzed in a cross-sectional study of workers exposed to CNT or CNF [26].

**Figure 10:**
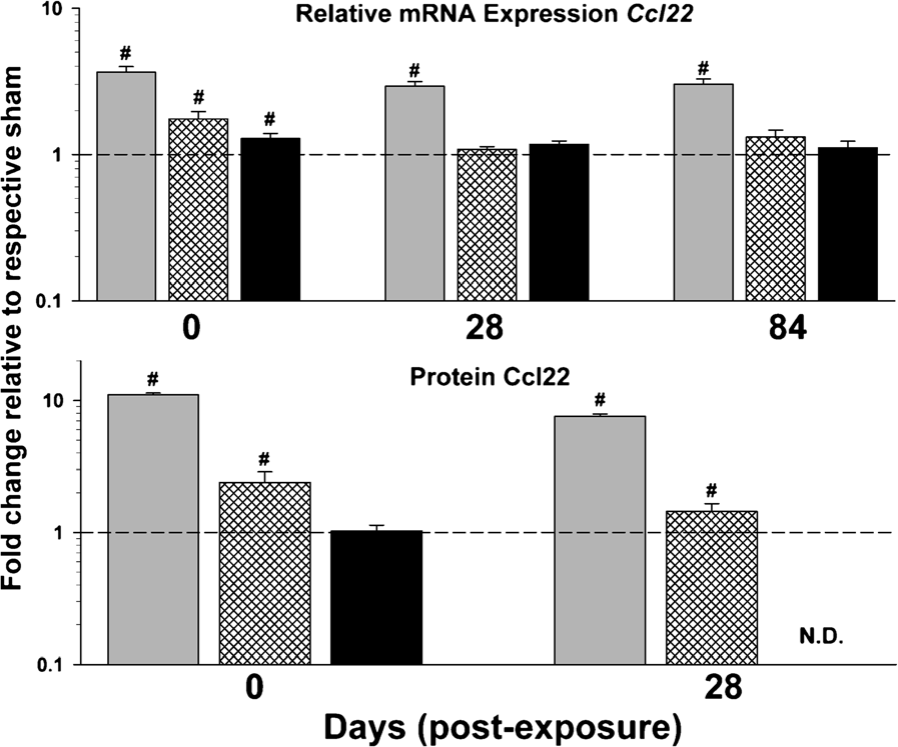
**Effect of multi-walled carbon nanotube (MWCNT) inhalation on pulmonary chemokine (C-C motif) ligand 22 (Ccl22) at different time points after exposure.** Pulmonary tissue gene expression (upper panel) and protein levels from the first fraction of collected bronchoalveolar lavage fluid were measured. Data are expressed as percent (%) change from respective sham (dashed line – 100%). Data presented as high dose (gray bars), medium dose (pattern), and low dose (black bars). N.D. – not determined. *p<0.05 vs. respective sham; #p<0.05 vs. other MWCNT depositions for a given time point only.

One mediator of interest was chemokine (C-C motif) ligand 22, also referred to as macrophage-derived chemokine. Studies involving first responders to 9/11 have shown that early increased serum CCL22/MDC correlated to declining pulmonary function in subsequent years [40]. *In vitro* studies showed that alveolar macrophages were the predominant producers of CCL22/MDC when compared to primary epithelial cells [41]. In a previous exposure study, pulmonary expression of *Ccl22*, with associated Ccl22 protein levels in the serum, were increased after a bolus dose of 40 μg MWCNT [8]. A more recent study confirmed those early findings of increased circulating Ccl22 [14]. Here, pulmonary expression levels were increased even at the lowest exposure dose (Figure 10), indicating the particular sensitivity of this mediator to MWCNT exposure in a mouse model. In addition to mRNA expression, protein levels in the BAL fluid were increased at the high and middle dose through 28 d post-exposure. Protein levels were not significantly increased at the lowest dose at 0 d. As future studies begin to uncover the molecular mechanisms associated with the pathology of MWCNT, especially at workplace relevant exposures, certain mediators, such as CCL22/MDC, may be more sensitive in determining exposure.

#### Pulmonary pathology of MWCNT

**Figure 11:**
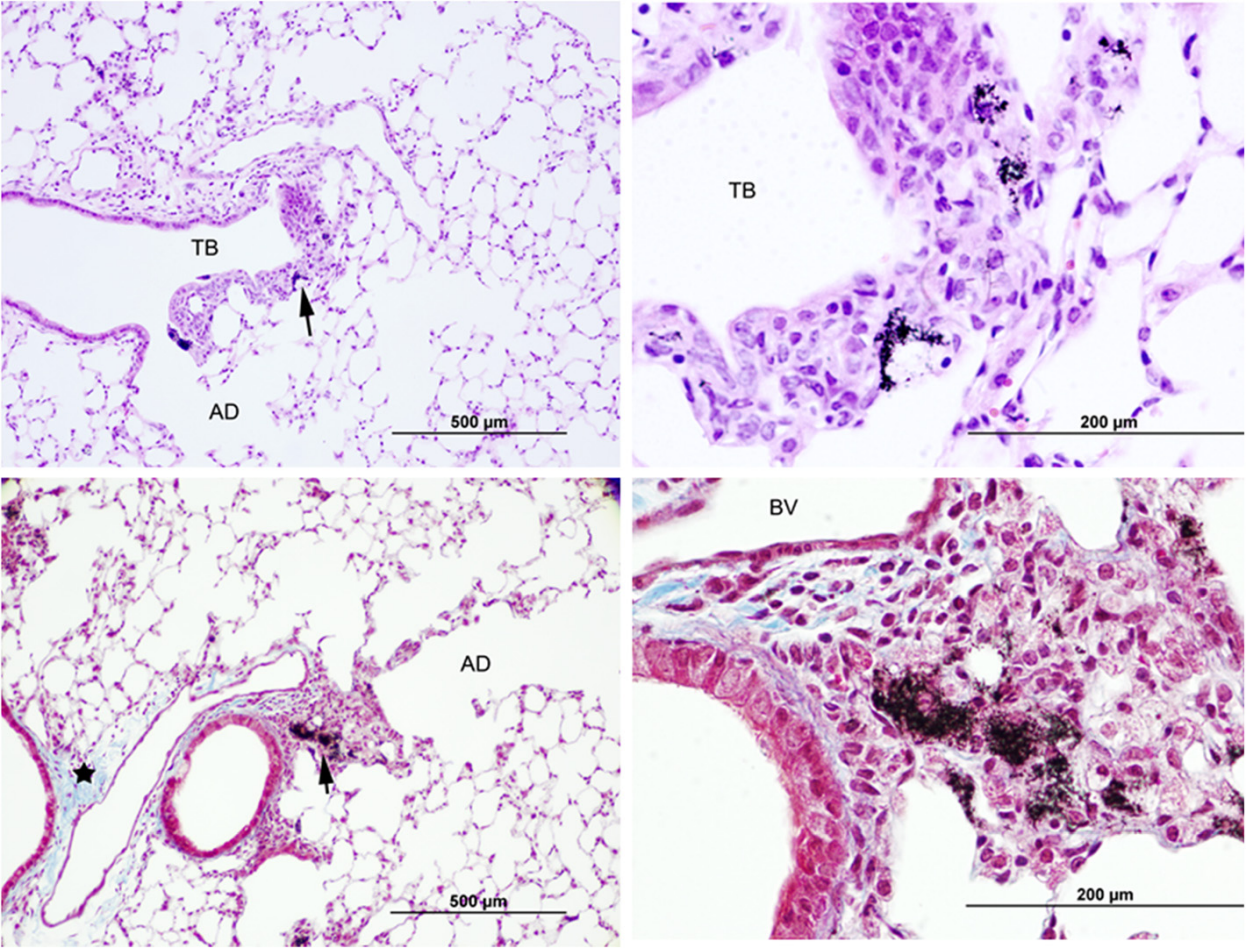
**Effect of multi-walled carbon nanotube (MWCNT) inhalation on pulmonary pathology.** Images are representative from mice exposed to the high dose (5 mg/m^3^ for 5 h/d for 19 d) and examined 84 d post-exposure. In the upper panels, MWCNT are within macrophages that have accumulated in connective tissue immediately below the thickened epithelium of the terminal bronchiole (TB). In comparison to the rest of the lung, general fibrosis, as identified by Trichrome staining, was scored as negative in MWCNT-treated mice (lower panels). No significant effects were scored for the middle and low dose exposures. The arrows in the left panels indicate the area of higher magnification shown in the respective right panel.

Mice tissues were harvested at 84 d post-exposure for pulmonary pathology. Only at the highest dose were changes indicated and included increased peribronchiolar inflammation and bronchiolar epithelial hyperplasia around the areas of MWCNT deposition with severity scored as minimal (1 on a 1–5 scale) in the MWCNT-exposed mice (Figure 11). Peribronchiolar inflammation incidence was 5/5 in MWCNT-exposed mice and 1/5 in air exposed shams and bronchiolar epithelial hyperplasia incidence was 5/5 in MWCNT-exposed mice and 0/5 in air exposed shams. As opposed to aspiration studies, there were no granulomas scored in this study, a finding consistent with inhalation using this particular MWCNT [5]. Overall fibrosis, determined solely from trichrome staining, was scored as negative. The findings are not unexpected as previous studies indicate the lack of pronounced diffuse pulmonary fibrosis at similar cumulative exposures [3]. Total collagen was not assessed in these studies and detailed morphometric analysis of the interstitium is underway. A previous study of 5 mg/m^3^ for 12 d, 7 d less exposure than the high dose from this study, showed a 53% increase in alveolar thickening by morphometric analysis [30]. While it is likely that detailed morphometric analysis would show evidence of interstitial fibrosis, the extrapolations to human relevancy suggest decades of continued exposure at an inhalable EC concentration of 10.6 μg/m^3^ may be necessary for substantial fibrosis to present. While risk was not defined in this study, the findings qualitatively support the recommended exposure limit predicting 0.5-16% risk of developing early-stage lung effects over a working lifetime (45 years) exposed to an 8 h time-weighted average of respirable EC concentrations of 1 μg/m^3^[28].

One effect that was not considered or measured in this study, or those evaluated in Figure 4, was the potential for carcinogenicity. Recent work by Sargent et al. showed that inhalation of MWCNT promoted mouse lung tumor formation following initiation by 3-methylcholanthrene [7]. The C x T for that study was 375 mg/m^3^ x h (5 mg/m^3^ x 15 d x 5 h/d) putting the cumulative exposure in the top tier of studies in Figure 4. While the data did not indicate initiation by MWCNT, they also did not show a no effect level for promotion. This was similar to *in vitro* studies that showed chromosomal effects were still measured at the lowest administered dose of 0.024 μg/cm^2^[42]. Therefore, a cutoff for extrapolated lung burden that would not result in tumor promotion cannot be currently determined, further indicating caution when handling CNT.

### Limitations

Several limitations were inherent to this study. One limitation was mass-based EC concentration cannot distinguish between other carbon species (e.g. diesel exhaust, burning of biomass, or other forms of nanomaterials such as graphene or fullerenes) which may over-estimate exposure to MWCNT. However, all efforts were attempted to minimize this by subtracting anthropogenic background concentrations of elemental carbon for each sample. For the human dosimetry calculation, we had to make several assumptions including using a fixed respirability to inhalability ratio to estimate the MMAD that was not specifically measured in the field. We used all of the the empirical data available to estimate an average deposition although it should be understood that variations in those parameters will affect alveolar deposition. Another limitation is the relatability of the animal exposure to the MWCNT found in workplace facilities. The MWCNT used in the animal inhalation study is a material that disperses very well as compared to many of the images observed from the PBZ samples. The inhalation material was also able to penetrate into the pleural space which is less likely for more agglomerated or thinner more flexible CNT [43]. Therefore, we believe that the toxicity for this particular exposure resides more in the worst case scenario category. To provide further insight, ongoing studies are evaluating toxicological endpoints of materials utilized by U.S. facilities.

## Conclusions

The cutoffs in Figure 4 were made in an arbitrary fashion from consensus findings across all studies. Discrepancies include the beginnings of histological aberrations at a lower dose [3], no PMN influx at any dose [10], and the transient LDH response soon after cessation of exposure at the middle dose of this study. In general, the recent findings from Ma-Hock et al. have increased pulmonary toxicity at lower cumulative C x T compared to other MWCNT inhalation studies depicted in Figure 4[2]. The differences between studies can be the result of different MWCNT structures produced by different inhalation system generations, differences in the types of MWCNT tested, timing of measurements, and the age, strain, and species used in each study. Irrespective, Figure 4 shows a relatively uniform prediction of effects for traditional measures of pulmonary toxicity when normalized to cumulative C x T with the assumption that a range exists for each specific cutoff depending on the type of MWCNT and inhalation exposure design. Recent studies have shown that specific functionalization of CNT can alter the toxicity [44-46]; it would be interesting to see how those materials would differ under similar inhalation exposure designs as previous studies.

The primary goal of this study was to provide context to relate exposure assessment studies in facilities manufacturing and handling MWCNT to dosimetry for *in vivo* rodent exposures. Exposure assessment data indicated an average inhalable EC concentration of 10.6 μg/m^3^ from PBZ measurements. Assuming a 25% inhalable to respirable ratio, the approximate respirable concentration is 2.65 μg/m^3^. It is clear from toxicological evaluations that MWCNT have a relatively high hazard when compared to other materials. These hazards may include fibrosis, promotion of lung tumors, cardiovascular dysfunction, and pulmonary and systemic inflammation. The present findings show that limiting cumulative exposures is imperative to reducing adverse effects.

## Methods

### Exposure assessment

A literature search was performed for exposure assessment studies at facilities handling MWCNT, SWCNT, or CNF in which personal breathing zone mass-based samples were collected. A total of 7 studies, over the past 10 yr, met the above criteria and are listed in Table 1. These studies represent a wide range of mass-based analysis methods, industries, and exposure scenarios all producing CNT/CNF or using the material downstream as a secondary manufacturer.

Exposure assessments were conducted at 8 different facilities producing or using MWCNT. Five of these facilities were previously reported [23] and an additional 3 MWCNT facilities were added to create an inhalable EC average from 8 facilities which were adapted to generate Figure 1. A detailed analysis of the findings from all facilities will be published separately (Dahm et al. [16], in preparation).

In brief, personal breathing zone, mass-based samples were collected to estimate the inhalable size fraction for EC. These samples were collected on open-faced, 25-mm-diameter quartz fiber filters (SKC Inc., Eighty Four, PA, USA) using Leland Legacy pumps (SKC Inc.) operating at 6–7 l min^-1^. The samples were subsequently analyzed according to NMAM Method 5040 [22] which is currently recommended by NIOSH to assess exposures to CNTs/CNFs [28]. Background samples for EC were collected in the same manner due to the potential interference from anthropogenic sources of EC. The background samples were collected as indoor or outdoor area samples based on the characteristics of each facility.

Concurrent, side-by-side personal breathing zone samples were also collected for transmission electron microscopy (TEM) to confirm the presence of MWCNT. Samples were collected on 25-mm mixed cellulose ester filters (0.8-lm pore size; SKC Inc.) using Leland Legacy pumps (SKC Inc.) operating at 5 l min^-1^. The TEM samples were then analyzed on a JEOL2100F TEM (JEOL USA, Inc., Peabody, MA, USA) using a modified NMAM 7402, asbestos by TEM [47]. Additional details on the sampling methodologies can be found in Dahm et al. [23].

### Study design and inhalation exposure in mice

Specific pathogen-free, male C57BL/6J mice from Jackson Laboratory (Bar Harbor, ME) were used in this study. All mice housed in the AAALAC-approved NIOSH Animal Facility were provided food and tap water *ad libitum* in ventilated cages in a controlled humidity and temperature environment with a 12 hr light/dark cycle. Animal care and use procedures were conducted in accordance with the “PHS Policy on Humane Care and Use of Laboratory Animals” and the “Guide for the Care and Use of Laboratory Animals” (NIH publication 86–23, 1996). These procedures were approved by the National Institute for Occupational Safety and Health Institutional Animal Care and Use Committee.

C57BL/6J mice, eight weeks of age, were exposed by inhalation to MWCNT (produced by Hodogaya, Japan) at various parameters to achieve two orders of magnitude range of lung burdens as shown in Table 2. Animals were exposed using a computer controlled whole body inhalation exposure system designed and constructed at NIOSH [48]. In brief, the inhalation exposure system combines air flow controllers, aerosol particle monitors, data acquisition devices, and custom software with automatic feedback control to achieve constant and repeatable exposure chamber temperature, relative humidity, pressure, aerosol concentration, and particle size distributions. The MWCNT used in this study have been extensively characterized previously [5,49]. The average diameter was 49 nm with a length of 3.86 μm (GSD 1.94). Purity was >99% carbon. Mice were exposed for 19 d over a total of 26 d (mice were not exposed for the three weekends and the second Monday of each four week exposure). The doses chosen were 5 mg/m^3^ for 5 h/d, 0.5 mg/m^3^ for 5 h/d, and 0.5 mg/m^3^ for 0.5 h/d. The lowest dose method was chosen because consistency was questioned in tests of exposure levels at 0.05 mg/m^3^. In this group, mice were exposed to air for 4.5 h then MWCNT for 0.5 h to maintain the same timeframe as the two other groups. Mice were euthanized at 0 d (immediately following the last exposure), 3 d, 28 d, and 84 d. The left lung lobe was ligated and frozen in liquid nitrogen and the right lung lobes were lavaged. Two separate groups were exposed for each dose for a total of n=12 air and n=12 MWCNT at each time point unless otherwise indicated. Mice in set one were used for histopathology (n=6 air; n=6 MWCNT) at 84 d post-exposure and mice in set two were used to include 3 d post-exposure (n=6 air; n=6 MWCNT).

### Lung burden of MWCNT

Previous studies have determined that male C57BL/6J mice exposed to 5 mg/m^3^ for 12 d have a total lung burden of 28.1 μg, or 2.34 μg/d [30]. In that same study, alveolar deposition was calculated at 84% of the total lung burden (or 1.97 μg/d). Assuming linearity, these values were extrapolated to achieve the lung burdens shown in Table 2. While these values are estimates because of the assumption of linearity, the values were generated from previously quantitated lung burdens in aged matched male mice of the same strain, C57Bl6/J [5,30].

### Bronchoalveolar lavage (BAL)

BAL collected from right lobes was assessed for lactate dehydrogenase activity, albumin concentration, and inflammatory protein concentrations. Differentials from the cellular fraction were made from counts of 300 cells per slide stained with Wright-Giemsa stain.

### Pulmonary gene expression

RNA was isolated from frozen lung using the RNeasy Mini Kit (Qiagen, Valencia, CA, USA). Evaluation of gene expression was determined by standard 96-well technology using the StepOne™ (Applied Biosystems, Carlsbad, CA, USA) with pre-designed Assays-on-Demand™ TaqMan® probes and primers including *Il6* (Mn00446190_m1), *Ccl2* (Mn00441242_m1), *Cxcl2* (Mn00436450_m1), and *Ccl22* (Mn00436439_m1) (Applied Biosystems). Using 96 well plates, one μg of total RNA was reverse transcribed using random hexamers (Applied Biosystems) and Superscript III (Invitrogen, Carlsbad, CA). Nine μl of cDNA (1/10) was then used for gene expression determination. Hypoxanthine-guanine phosphoribosyltransferase was used as an internal reference. Relative gene expression was calculated using the comparative threshold method (2^-ΔΔCt^) with vehicle-treated mice serving as the reference group [50].

### BAL protein analysis

Collected acellular first fraction BAL was sent to Myriad / RBM (Austin, TX) for protein profiling by multiplex immunoassay RodentMAP v3.0. Only select proteins were used for illustration in the manuscript and the complete analysis will be published in a subsequent manuscript.

### Histopathology

Right lung sections were cut, stained, and sent for histopathology assessment by Charles River Research Animal Diagnostic Services. Histopathology that was assessed included peribronchiolar inflammation, bronchiolar epithelial hyperplasia, and fibrosis. Sections were scored on a 1–5 scale where 1=minimal and 5=severe.

### Statistics

Statistical analysis of all outcome variables was performed using SAS version 9.3 for Windows. The exposure assessment PBZ EC concentrations were calculated, specific to each facility, by subtracting the area EC background data from the PBZ data. This was done to separate any anthropogenic EC sources from potential CNT/CNF EC exposures. Any samples that were below the limit of detection (LOD) for method 5040 were calculated by taking the LOD of the method and dividing it by two and then calculating the air concentration using the volume of that specific sample. These data were used to calculate the arithmetic means and SD. In order to calculate the geometric means and GSDs, the data were log transformed. The normality of the log-transformed data were assessed using the Shapiro-Wilk test.

Data from rodent studies were log transformed prior to analysis to meet the assumptions of the statistical tests. roc Mix was used to run a three-way factorial analysis of variance. Significant three-way interactions were examined by utilizing two-way ANOVA’s stratified by time. Pairwise comparisons were performed using Fishers Least Significant Difference test. Differences between experimental groups were considered significant with p-values less than 0.05.

## Competing interests

The authors declare that they have no competing interests.

## Authors’ contributions

AE and MD conceived and designed the study and drafted the manuscript. MLK and JAD conducted the statistical analysis. MEB and DEE conducted PBZ sample analysis. BTC, MKSB, HDL, WM, DGF, JMA, DWP, and VC provided insight and conducted the mouse inhalation exposures. AE, PCZE, SAB, TH, LB, JEF, and DSB did electron microscopy, gene and protein determinations, histology preparation, and measured markers of pulmonary injury from collected bronchoalveolar lavage. All authors read and approved the final manuscript.
